# A patient with Pfeifer-Weber-Christian Disease - Successful Therapy with Cyclosporin A: case report

**DOI:** 10.1186/1471-2474-11-18

**Published:** 2010-01-27

**Authors:** Georg Pongratz, Boris Ehrenstein, Wolfgang Hartung, Jürgen Schölmerich, Martin Fleck

**Affiliations:** 1 Dept. of Internal Medicine I, University Medical Center Regensburg, 93042 Regensburg, Germany; 2 Dept. of Rheumatology/Clinical Immunology, Asklepios-Clinic, 93077 Bad Abbach, Germany

## Abstract

**Background:**

Pfeifer-Weber-Christian disease (PWCD) is a rare inflammatory disorder of the subcutaneous fatty tissue. The diagnosis and therapy of this rare type of panniculitis is still controversial and will be discussed in this article.

**Case presentation:**

We here report the rare case of a 64-year old male patient, with PWCD. The patient suffered from rheumatoid arthritis for several years, but then developed relapsing fever and recently occurring painful subcutaneous nodules predominantly at the inner part of his left upper limb with no signs of synovitis. Finally, a biopsy from one of the nodules revealed lobular panniculitis with mixed cell infiltrate, which was conformable only with PWCD, after excluding several differential diagnoses. In our patient PWCD developed despite immunosuppressive therapy with steroids and different disease modifying drugs, which the patient received to treat his underlying rheumatoid arthritis. However, when DMARD therapy was switched to Ciclosporin A the patient's symptoms resolved.

**Conclusion:**

Our observation supports the hypothesis that T cells are involved in the pathogenesis of PWCD. Thus, T cell modifying drugs should be primarily used to treat patients with this rare disorder.

## Background

Pfeifer-Weber-Christian Disease (PWCD) is a rare inflammatory disorder of subcutaneous adipose tissue. It is also known as ideopathic relapsing febrile lobular non-suppurative panniculitis and is characterized by recurrent subcutaneous inflammatory painful nodules, fever, and malaise due to systemic inflammation. PWCD is an exclusion diagnosis, when no other cause of the lobular panniculitis can be identified. The most important other causes of lobular panniculitis include systemic lupus erythematosus, α1-antitrypsin deficiency, lymphoma, trauma, pancreatitis, and certain types of infections (for review see [1,2]). The characteristic painful nodules are observed primarily in the area of the lower extremities and the trunk. It is still debated if PWCD exists as a unique disease entity or if it is just a substitute for any lobular panniculitis with unknown cause. The reason for this debate is that, having more medical knowledge and better diagnostic tools, many of the cases of idiopathic lobular panniculitis diagnosed with so-called PWCD have other underlying causes [1,3,2]. However, we here present the case of a 64-year old male patient with relapsing febrile lobular non-suppurative panniculitis with mixed cell infiltrate of unknown origin. Even after extensive diagnostic procedures we could not identify any of the known causes of lobular panniculitis. We therefore diagnosed a PWCD and successfully treated the patient with Cyclosporin A. The case supports the hypothesis that PWCD is a T cell mediated autoinflammatory condition.

## Case presentation

Here, we report the rare case of a 64-year old male patient with rheumatoid arthritis, relapsing fever and recently occurring painful subcutaneous nodules predominantly at the inner part of his left upper limb.

**Figure 1:**
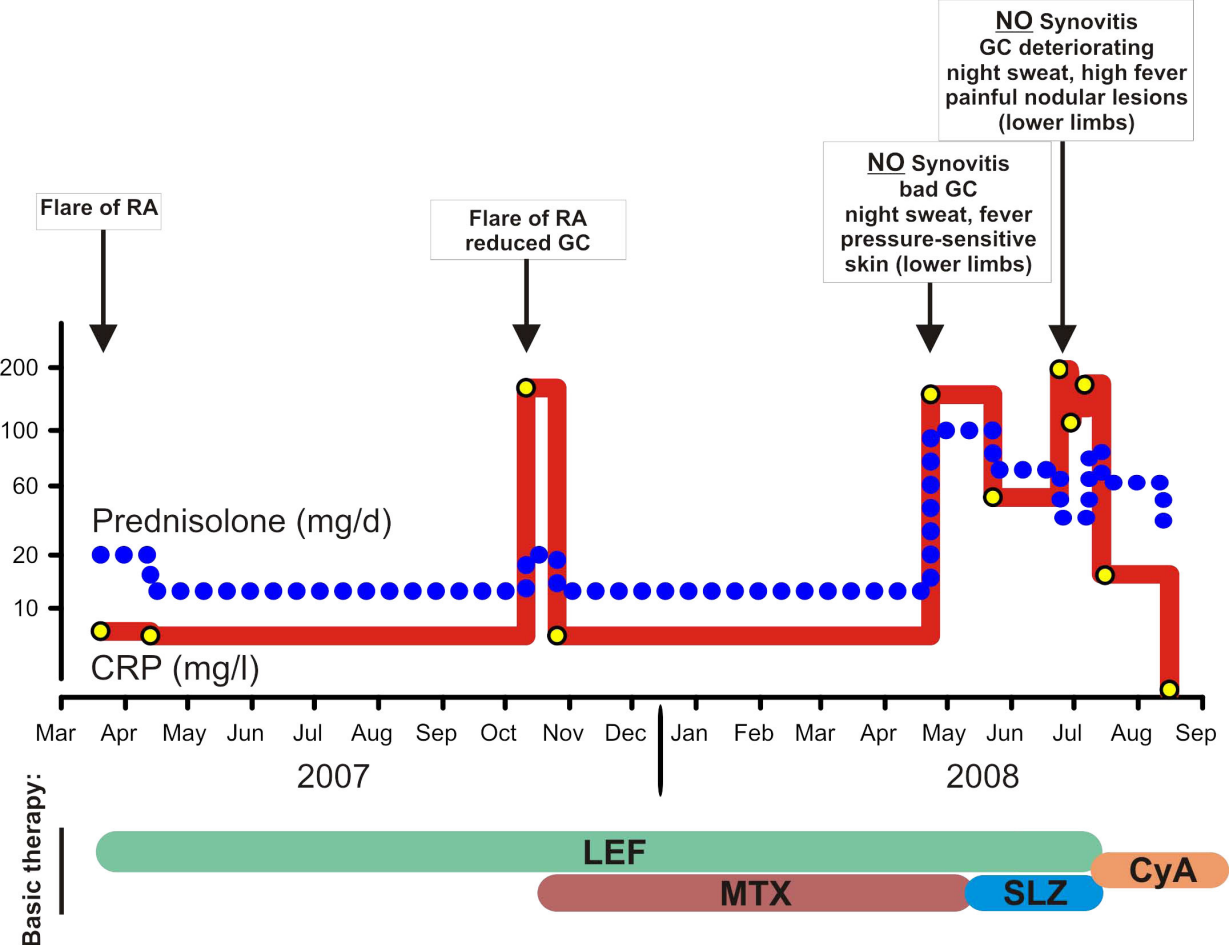
**Schematic time course of C-reactive protein levels and concurrent therapy**. Dashed-line: Prednisolone (mg/d); solid-line: CRP (mg/l). CRP: C-reactive protein; RA: rheumatoid arthritis; GC: general condition; LEF: leflunomide; MTX: methotrexate; SLZ: sulfasalazine; CyA: cyclosporine A.

**Figure 2:**
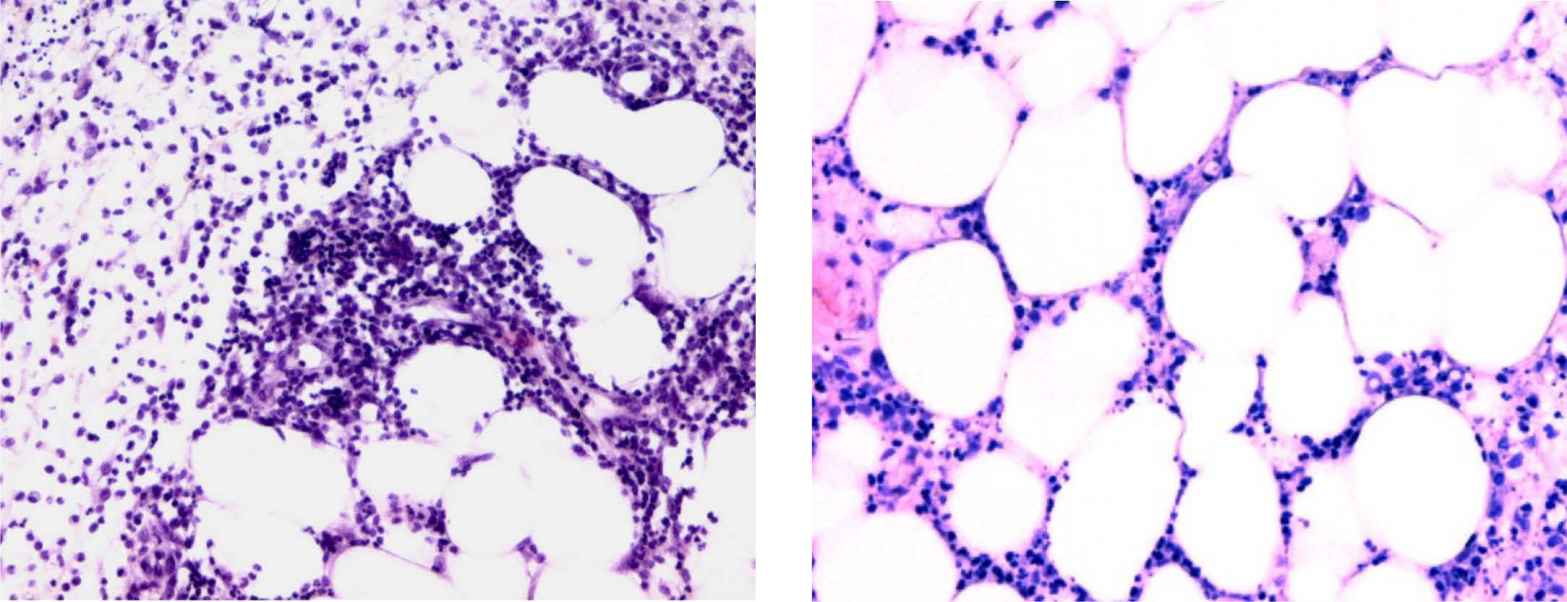
**Histology (painful node, left lower leg)**. Standard histological processing was performed on a painful node obtained from the lower limb. Two HE-stained sections at different magnifications (left 20×, right 30×) are depicted. The sections show predominantly lobular panniculitis with mixed inflammatory cell infiltration.

On first encounter with the patient in 2007, he reported symptoms of arthritis with varying intensity since 1992. However, till 2003 he did not receive any specific therapy for his symptoms. In 2003 he was diagnosed with RF positive, anti-CCP antibody negative rheumatoid arthritis (RA), which was initially treated with methotrexate (MTX) and prednisolone. However, therapy with MTX was discontinued after a few weeks due to unspecific malaise. In early 2007, the patient was referred to our Department with an acute flare of RA (Figure 1). The acute symptoms were successfully treated with prednisolone. Additional disease modifying anti-rheumatic drug (DMARD) therapy with leflunomide was initiated, which was well tolerated by the patient, except for mild edema of his lower legs. About half a year later, the patient presented again with highly elevated humoral inflammatory parameters (Figure 1) and pain on exertion in both hips, knees and ankles with swelling of the latter two as well as several MCP joints. At this time, there were no fever or night sweats, and the patient had gained 10 kg of body weight after his last admission. Again, prednisolone treatment was started (max. 20 mg/d) leading to rapid improvement. In addition, low dose subcutaneous MTX therapy was added to leflunomide. On routine check-up after 6 month, the patient had no joint pain or swelling and no signs of synovitis by ultrasonography. However, humoral inflammatory activity was still high (Figure 1), and the patient described his general condition as "deteriorating" with fever, night sweats and loss of body weight. He additionally complained about paresthesia and pain on pressure in both feet and lower legs. Despite comprehensive diagnostic evaluation including echocardiography, sonography of joints, soft tissue and abdomen, computed-tomography of the thorax, repeated blood cultures, bone marrow aspiration and urine testing, we were not able to identify any infectious or malignant cause for the patients deterioration. Also, values for ANA, ANCA, anti-ds-DNA, complement factors C3 and C4, anti-CCP, and anti-MCV were within the normal range or not detectable, respectively. RF was positive, but has been positive since the diagnosis of RA in 2003. Due to extremely pressure sensitive skin of the lower limbs skin-biopsies were obtained, which revealed an unspecific chronic fibrosing inflammation. Furthermore, a positron emission tomography with fluorodeoxyglucose (FDG-PET) was performed, which demonstrated focal areas of increased glucose uptake on the lower limbs but did not show a typical vasculitis pattern. On a repeated thorough clinical examination, new small (< 5 mm) subcutaneous nodules could be palpated mainly at the inner side of the thighs. However, those lesions could not be detected by ultrasonography. Thus, no biopsy was taken at that time. Treatment with MTX was stopped and replaced by sulfasalazine (Figure 1). Following treatment with high dose prednisolone, the patient's condition improved significantly and CRP levels decreased rapidly (Figure 1). However, one month later the patient presented in deteriorated condition with high inflammatory parameters (CRP 220 mg/l, Figure 1). He presented with high fever (up to 40°C), lasting for several days, night sweats and increasing painful nodular lesions (3 - 4 cm) at the inner side of the thighs that led to a complete immobilisation of the patient due to pain. On first sight, the lesions appeared as an erythema nodosum, only with less apparent skin discoloration. With regard to the joints, the patient had no complaints and physical examination revealed no signs of synovitis. One of the painful nodules was removed for histological analysis. An MRI-scan of the lower limbs revealed signs of acute fasciitis. After exclusion of any infectious or malignant cause, the patient was again treated with high dose prednisolone (80 mg/d, i.v.). The histology of the nodule revealed a lobular panniculitis, with a mixed cell infiltrate dominated by neutrophils (Figure 2). Together with the clinical findings with a strong systemic inflammatory response without any indication of a flare of the rheumatoid arthritis or underlying infectious or malignant cause, we established the diagnosis of a Pfeifer-Weber-Christian disease (PWCD). Leflunomide and sulfasalazine were therefore replaced by cyclosporine A. The patient responded well to this treatment and temperature as well as inflammatory markers returned back to normal levels rapidly (Figure 1). The patient could be discharged from the hospital. On a routine check-up in the following month the patient presented in very good general condition with no more signs of inflammation. The nodules were still present but clearly reduced in size, and the patient only reported minor pain on pressure.

## Discussion

Here, we report the rare case of a Pfeifer-Weber-Christian syndrome, which responded well to therapy with prednisolone and cyclosporine A.

The diagnosis of a Pfeifer-Weber-Christian disease (PWCD) is depending on the presence of relapsing fever, systemic inflammation and panniculitis. However, due to the rarity of this condition it is still under debate, if PWCD is a unique disease entity, or if it is just a substitute for every panniculitis that does not fit a common diagnosis [3]. This uncertainty might be the result of the fact that this disease entity has not been described in a monolithic way, and the original cases reported by Pfeifer, Weber and Christian differed slightly from each other [4]. Pfeifer in 1892 first described multiple areas of atrophy in subcutaneous fatty tissue with no further specification [4]. Weber described a "relapsing, non-suppurative panniculitis" in 1925 [5], and Christian in 1928 pointed out its febrile character [6]. However, this can also be regarded as a process of refining symptoms resulting in the symptom complex of fever, systemic inflammation and lobular panniculitis of relapsing character, to finely define PWCD. With respect to the histological findings in panniculitis, an attempt was made to categorize nodular panniculitis into different histologically defined subtypes according to the pattern and composition of the inflammatory infiltrate [7]. PWCD has been classified as lobular panniculitis with mixed cell infiltrate, which contrasts findings in erythema nodosum classified as predominantly septal panniculitis [7].

Unfortunately, in several reported cases the diagnosis of PWCD was solely based on histological findings [3]. However, in our opinion diagnosis should always be based on clinical and histological findings, which might facilitate the recognition of PWCD as unique disease entity. Of course, the described symptoms together with lobular panniculitis can also occur in other diseases such as infections, certain malignancies, alpha-1-antitrypsin deficiency, pancreatitis, systemic lupus erythematosus and cytophagic histiocytic panniculitis, rendering PWCD an exclusion diagnosis following careful evaluation of the patient. However, all of these disease entities, except alpha-1-antitrypsin deficiency, were excluded in our patient by extensive diagnostic evaluation favouring the diagnosis of a PWCD (see above). We did not explicitly exclude alpha-1-antitrypsin deficiency, because panniculitis is only observed in patients with severe forms of the disease [2,8], which would include other symptoms, like COPD and/or liver cirrhosis. However, our patient didn't show these symptoms and responded well to immunosuppressive therapy, which would not improve panniculitis due to alpha-1-antitrypsin deficiency.

Treatment of PWCD is also controversial, which might again be attributed to the fact that clear diagnostic criteria have not yet been established. Thus, it does not surprise that there are several treatment options reported in the literature mostly in the form of case reports. However, the best evidence exists for cyclosporine A and corticosteroids [9-17]. Our patient improved rapidly following cyclosporine A and corticosteroid treatment. Cyclosporine A is classified as a calcineurin inhibitor predominantly inhibiting IL-2 production and transduction of antigen-recognition signals in activated T cells [18]. In the here reported case, leflunomide, methotrexate and sulfasalazine, despite displaying a broader range of immunosuppressive activity by interacting with multiple signaling pathways in various cell types, were not effective [18]. Thus, even though the pathogenesis of PWCD is not finally resolved, the fact that the patient only responded to cyclosporine A points towards a predominant involvement of T-cells in the disease process.

## Conclusion

In conclusion, this case report supports the hypothesis that PWCD is a T-cell mediated inflammatory disorder. Therefore, in our opinion, patients with PWCD should primarily be treated with T-cell modifying agents. However, it is still controversial if PWCD exists as a separate disorder or if the characteristic symptoms and inflammatory patterns are just a manifestation of an other underlying cause. Therefore, consideration of PWCD has to be based on a broad diagnostic procedure to exclude all likely differential diagnoses of lobular panniculitis, like infections, certain malignancies, alpha-1-antitrypsin deficiency, pancreatitis, systemic lupus erythematosus and cytophagic histiocytic panniculitis.

## Competing interests

The authors declare that they have no competing interests.

## Authors' contributions

GP data aquisation, data interpretation, manuscript preparation; BE data aquisation, manuscript preparation; WH data aquisation, substential manuscript revision; JS substential manuscript revision, data interpretation; MF substential manuscript revision; data interpretation;

All authors read and approved the final manuscript.

## Patient's consent

Written informed consent was obtained from the patient for publication of this case report and accompanying images. A copy of the written consent is available for review by the Editor-in-Chief of this journal.

## Pre-publication history

The pre-publication history for this paper can be accessed here:

http://www.biomedcentral.com/1471-2474/11/18/prepub

## References

[B1] Michael P Gratiot J Mesenteric panniculitis associated with trauma Calif Med 1964 100 285 287 14165879 PMC1515532

[B2] Requena L Sanchez YE Panniculitis. Part II. Mostly lobular panniculitis J Am Acad Dermatol 2001 45 325 361 10.1067/mjd.2001.114735 11511831

[B3] White JW Jr Winkelmann RK Weber-Christian panniculitis: a review of 30 cases with this diagnosis J Am Acad Dermatol 1998 39 56 62 10.1016/S0190-9622(98)70402-5 9674398

[B4] Pfeifer V Über einen Fall von herdweiser Atrophie des subkutanen Fettgewebes Deutsches Archiv für klinische Medizin 1892 438 449

[B5] Weber FP A case of relapsing non-suppurative nodular panniculitis, showing phagocytosis of subcutaneous fat-cells by macrophages British Journal of Dermatology and Syphilis 1925 301 311 10.1111/j.1365-2133.1925.tb10003.x

[B6] Christian HA Relapsing febrile nodular nonsuppurative panniculitis Archives of Internal Medicine 1928 338 351 10.1001/archinte.1956.00250190055002 13275145

[B7] Niemi KM Forstrom L Hannuksela M Nodules on the legs. A clinical, histological and immunohistological study of 82 patients representing different types of nodular panniculitis Acta Derm Venereol 1977 57 145 154 71810

[B8] Smith KC Pittelkow MR Su WP Panniculitis associated with severe alpha 1-antitrypsin deficiency. Treatment and review of the literature Arch Dermatol 1987 123 1655 1661 10.1001/archderm.123.12.1655 3318708

[B9] Iwasaki T Hamano T Ogata A Successful treatment of a patient with febrile, lobular panniculitis (Weber-Christian disease) with oral cyclosporin A: implications for pathogenesis and therapy Intern Med 1999 38 612 614 10.2169/internalmedicine.38.612 10435371

[B10] Kovacs M Hafner J Gabor V [Successful treatment of Weber-Christian panniculitis with cyclosporin-A] Orv Hetil 2004 145 827 831 15188638

[B11] Hinata M Someya T Yoshizaki H Successful treatment of steroid-resistant Weber-Christian disease with biliary ductopenia using cyclosporin A Rheumatology (Oxford) 2005 44 821 823 10.1093/rheumatology/keh576 15757971

[B12] Miyasaka N Steroid-resistant Weber-Christian Disease Intern Med 1999 38 522 10.2169/internalmedicine.38.522 10435355

[B13] Viravan S Wisuthsarewong W Manonukul J Successful treatment of cytophagic histiocytic panniculitis by cyclosporin A: a case report Asian Pac J Allergy Immunol 1997 15 161 166 9438549

[B14] Royle G Blacklock H Miller M Treatment of cytophagic panniculitis with cyclosporin A Am J Med 1992 92 704 705 10.1016/0002-9343(92)90794-C 1605157

[B15] Usuki K Kitamura K Urabe A Successful treatment of Weber-Christian disease by cyclosporin A Am J Med 1988 85 276 278 3400710 10.1016/s0002-9343(88)80368-1

[B16] Entzian P Barth J Monig H Treatment of Weber-Christian panniculitis with cyclosporine A Rheumatol Int 1987 7 181 10.1007/BF00270368 3671993

[B17] Ostrov BE Athreya BH Eichenfield AH Successful treatment of severe cytophagic histiocytic panniculitis with cyclosporine A Semin Arthritis Rheum 1996 25 404 413 10.1016/S0049-0172(96)80005-9 8792512

[B18] Allison AC Immunosuppressive drugs: the first 50 years and a glance forward Immunopharmacology 2000 47 63 83 10.1016/S0162-3109(00)00186-7 10878284

